# Ethical Decision-Making in Education: A Comparative Study of Teachers and Artificial Intelligence in Ethical Dilemmas

**DOI:** 10.3390/bs15040469

**Published:** 2025-04-04

**Authors:** Neslihan Karakuş, Kerim Gedik, Semin Kazazoğlu

**Affiliations:** 1 Department of Turkish Language Education, Yıldız Technical University, Istanbul 34220, Turkey; nkarakus@yildiz.edu.tr; 2 Foreign Languages Education Department, Faculty of Education, Yıldız Technical University, Istanbul 34220, Turkey; semink@yildiz.edu.tr

**Keywords:** decision-making, artificial intelligence (AI), ethical dilemmas, teacher of ethics, teacher education

## Abstract

The use of artificial intelligence (AI) in education supports long-term educational goals. AI enables learners to engage with ethical issues through simulations and virtual environments, allowing them to experience responsible decision-making in practice. Additionally, it assists administrators and educators in making data-driven decisions, contributing to the more effective formulation of educational policies. This study examines how teachers and AI address ethical educational dilemmas, comparing their decisions based on gender, experience, and education level. A total of 141 public school teachers in Turkey participated in the study, and their responses were compared with AI-generated decisions using Yin’s nested multiple-case design. The scenarios were analyzed within the framework of five ethical perspectives: virtue ethics, deontological ethics, utilitarianism, social justice ethics, and situation ethics. AI aligned with teachers in five out of eight ethical dilemmas but differed in three cases, adopting a more analytical and outcome-oriented approach. In contrast, teachers placed greater emphasis on empathy and adherence to ethical principles. These findings highlight the differences in ethical decision-making between AI and teachers, emphasizing the role of AI in fostering ethical responsibility and awareness in education.

## 1. Introduction

Technological advancements and artificial intelligence (AI) profoundly influence the decision-making processes of individuals, institutions, and societies, becoming an integral part of daily life. With capabilities such as big data analysis, predictive modeling, and process automation, AI enhances efficiency across various fields by offering innovative solutions (Reid & Petocz, 2006). Increasingly adopted in education, healthcare, and business, these systems transform decision-making processes and position the human factor as a complementary element (Öztemel, 2020). Particularly in the field of education, AI supports teachers and administrators in decision-making, significantly contributing to educational management and instructional processes (Hwang et al., 2020; Ince et al., 2021; Pradeep Kumar et al., 2023).

In education, AI not only provides technical support to teachers but can also play a decisive role in ethical decision-making processes (Bozkurt, 2023). While AI’s data-driven analysis capability helps educators make more informed and consistent decisions, its alignment with human values such as empathy and fairness in ethically sensitive situations remains debatable (Kassymova et al., 2023). In this context, it is crucial to examine how teachers and AI systems handle ethical dilemmas and how these processes influence educational decision-making mechanisms.

### 1.1. Ethical Decision-Making Approaches

To understand ethical decision-making processes, it is necessary to first address the fundamental principles of ethics. As emphasized by Pieper (1999), ethics focuses on human actions, particularly moral decision-making. As one of the four main branches of philosophy—alongside epistemology, ontology, and logic—ethics is recognized as a field directly related to human nature (Iyi & Tepe, 2011). Ethical thought has evolved around different approaches throughout history, serving as fundamental principles that shape individual and societal decision-making processes. In this framework, the question of how AI can be integrated into ethical decision-making mechanisms and the extent to which it aligns with teachers’ ethical sensitivities emerges as a critical research issue. While AI-based systems make decisions based on objective data, teachers rely on pedagogical intuition and ethical values in their decision-making processes. This distinction raises the question of which approaches are more effective in addressing ethical dilemmas in education.

Historically, two main ethical approaches have been prominent. The “utilitarian approach”, developed by Bentham and Mill, argues that the moral value of an action should be measured by its benefits, whereas Kant’s “deontological ethics approach” prioritizes the nature and intent of the action (Ismail & Benlahcene, 2018; Iyi & Tepe, 2011). When examining the role of these approaches in educational ethical dilemmas, utilitarianism appears to be effective in pragmatic decision-making processes, whereas deontology takes precedence in moral obligation-based decision-making.

Beyond traditional ethical understandings, modern ethical approaches also play a significant role at the intersection of education and technology. For instance, the “situational ethics” perspective suggests that the moral value of an action depends on its context and may vary according to circumstances. Similarly, Rawls’ “ethics of justice” model focuses on constructing a fair social structure that protects individual rights (Nagel, 1973). Within this framework, ethical dilemmas in education are shaped not only by theoretical models but also by the practical decisions of educators and technological systems. These different ethical perspectives are summarized in Table 1, which categorizes major ethical approaches, their defenders, historical periods, and key principles.

### 1.2. Ethical Dilemmas in Education: A Comparison Between Teachers and AI

AI is increasingly seen as a tool for addressing complex ethical dilemmas (Akgun & Greenhow, 2022), particularly in fields where ethical sensitivity is critical, such as education (Al-kfairy et al., 2024). However, given that human ethical values are internalized through intuitive and empathy-based approaches, the extent to which AI can successfully model these processes remains debatable. Therefore, the similarities and differences between teachers’ ethical decision-making mechanisms and AI systems’ responses to ethical dilemmas should be examined. Through this comparative analysis, a more holistic perspective on ethical decision-making processes in education can be developed.

With technological advancements, artificial intelligence (AI) plays an increasingly active role in various aspects of daily life. However, its integration into these processes also raises significant ethical debates. Fundamental ethical principles such as autonomy, justice, and non-malfeasance bring forth questions about how AI should be guided and how well it aligns with human-centered ethical frameworks (Prakash et al., 2022).

The fact that algorithms are designed by humans has sparked a crucial debate regarding whether AI can be ethically neutral. Consciously or unconsciously encoded biases raise the possibility that AI-driven decision support systems may either reinforce or weaken ethical values (Guan et al., 2022). In the field of education, AI is seen as a tool that can support teachers’ ethical decision-making. However, uncertainties persist regarding the extent to which AI can make decisions based on emotional and human values (Kassymova et al., 2023). While AI systems can assist teachers in decision-making processes, it remains unclear how well they can comprehend emotional dimensions and ethical frameworks.

Different perspectives exist on whether AI can bear ethical responsibility. Legal regulations and societal acceptance processes are key factors that determine AI’s role in decision-making mechanisms (Burukina et al., 2019; Malyshkin, 2015), while risks such as ethical inequalities and algorithmic biases in education must also be taken into account (Kassymova et al., 2023; Li et al., 2019). Some researchers argue that AI systems developed in alignment with ethical principles can contribute to decision-making processes across various domains (Çelebİ, 2019). However, it is also argued that allowing AI to act entirely independently could lead to uncontrollable consequences (Kaku, 2011), making a model where final decision-making responsibility remains with humans a safer approach (Abel et al., 2016).

In the field of education, the ethical dilemmas faced by teachers serve as an important reference point for understanding the extent to which AI can be integrated into decision-making processes (Bucholz et al., 2007; Koç, 2010; Levent & Şall, 2022). Ethical dilemmas arise as teachers encounter unethical demands, student behavioral issues, and school policies (Helton & Ray, 2006). Teachers’ decision-making processes within a professional ethical framework are reinforced through reflective practices and peer support (Ehrich et al., 2011; Feng, 2007). However, the extent to which ethical dimensions are prioritized in teacher training programs remains a subject of debate.

Studies on teachers’ ethical decision-making processes highlight the strong connection between professional knowledge and ethical approaches. Lyons (1990) emphasized the importance of reflective practices in helping teachers navigate ethical and epistemological dilemmas during their knowledge acquisition processes. Husu (2001) examined the conflicts between teachers’ professional responsibilities and personal values, exploring strategies to resolve these dilemmas. Nakar (2017) analyzed how conflicts between economic interests and professional ethics affect teachers’ job satisfaction. Similarly, Gierczyk & Harrison (2019) evaluated how teachers in inclusive classrooms balance meeting individual student needs with the principle of justice. These studies underscore the need to enhance ethical sensitivity in teacher training programs.

When comparing teachers’ ethical decisions with AI-driven decision-making mechanisms, fundamental differences become apparent. While AI systems base their decisions on predefined algorithms (Abel et al., 2016), teachers rely on personal experience, empathy, and human values (Ehrich et al., 2011). AI can make systematic decisions using big data analytics and statistical approaches, whereas teachers adopt a more holistic perspective by considering individual differences, classroom dynamics, and emotional contexts in their decision-making processes. This highlights the necessity for AI to incorporate a human-centered approach to ethical decision-making.

The integration of AI technologies in education offers significant opportunities for both teachers and students (Dieterle et al., 2024; Park & Kwon, 2024). Tools like ChatGPT 4o facilitate lesson planning, provide instant feedback to students, and simplify assessment processes (Adiguzel et al., 2023). However, the widespread use of these technologies also introduces ethical and pedagogical risks (Karakose, 2023). The misuse of AI by students poses potential risks regarding academic integrity and critical thinking skills (Adiguzel et al., 2023). Assignments completed entirely by AI could weaken learning processes and negatively impact academic development (Karakose, 2023). Additionally, the possibility of AI generating misleading information presents challenges for both students and teachers in accessing reliable knowledge (Su & Yang, 2023). Therefore, it is crucial to use AI tools consciously and integrate them into the education system in a balanced manner.

In recent years, research on AI ethics has increased significantly (Akgun & Greenhow, 2022; Holmes et al., 2022). However, studies comparing teachers’ ethical decisions with those made by AI remain limited (Casas-Roma et al., 2021). Addressing this research gap could contribute to a more conscious and balanced integration of AI in education. This study aims to compare teachers’ and AI’s approaches to ethical dilemmas, identifying similarities and differences between the two. By examining ethical theories, the study seeks to analyze the extent to which teachers’ and AI’s ethical decision-making processes align or diverge.

In particular, this study seeks answers to the following question:

1. What is the relationship between the decision-making processes given by teachers and artificial intelligence in ethical dilemma scenarios?

## 2. Materials and Methods

### 2.1. Research Design

This study employs a nested multiple-case design (Yin, 2017) to compare teachers’ and artificial intelligence (AI) approaches to ethical dilemmas. This design enables a detailed and context-based examination of multiple cases and their subunits, making it suitable for studies that require in-depth analysis of complex decision-making processes. In this research, teachers and AI systems were defined as the main cases, while their responses to different ethical dilemma scenarios served as subunits of analysis.

The ethical dilemmas were categorized under specific themes and answered by both teachers and the AI system. To ensure a consistent basis for comparison, similar conditions were established for both cases. Each scenario was further subdivided based on responses from different teacher groups and AI outputs, allowing for a nuanced analysis of ethical perspectives and reasoning patterns.

The selection of Yin’s (2017) nested multiple-case design was based on the need for a methodology that supports in-depth and contextual comparison of two fundamentally different decision-making systems—human and artificial. Since the aim of the study was not only to document the decisions made but also to understand the reasoning behind those decisions, a qualitative approach that allows for rich data collection and analysis was essential.

Alternative research designs, such as experimental or survey-based methods, were considered during the design phase. However, experimental designs were found to be limited in capturing the complexity and contextual nature of ethical reasoning, as they tend to focus on predefined variables and controlled conditions. Similarly, survey methods could provide generalizable data but would not offer sufficient depth to explore the underlying justifications and thought processes guiding ethical decisions. The nested multiple-case design, on the other hand, provided the flexibility to examine each case comprehensively while maintaining a comparative perspective. In this study, the ethical decision-making processes of teachers were analyzed in depth first. Once this phase was completed, the responses of the AI system were examined separately. This sequential structure facilitated an in-depth examination of each case, ensuring a systematic approach to analysis while capturing the distinct features of each context comprehensively.

By structuring the study in this way, the similarities and differences between teachers and AI’s ethical decision-making mechanisms were evaluated comprehensively. This approach enabled the identification of both contextual and structural patterns in ethical reasoning, offering valuable insights into how ethical dilemmas are approached by human educators and artificial intelligence systems.

### 2.2. Participants

**Figure 1:**
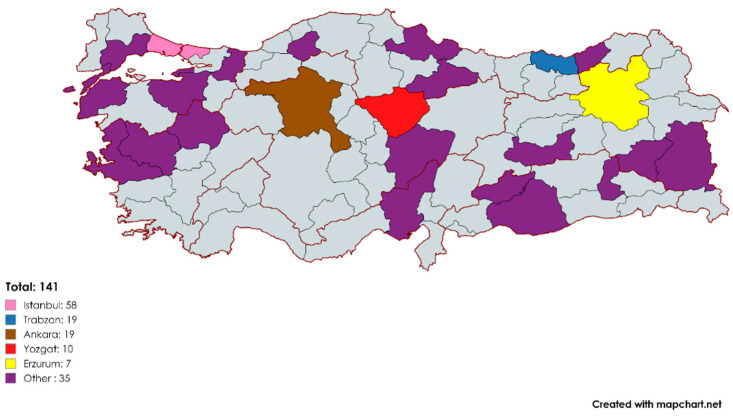
Study participant demographics (MapChart, 2025).

**Figure 2:**
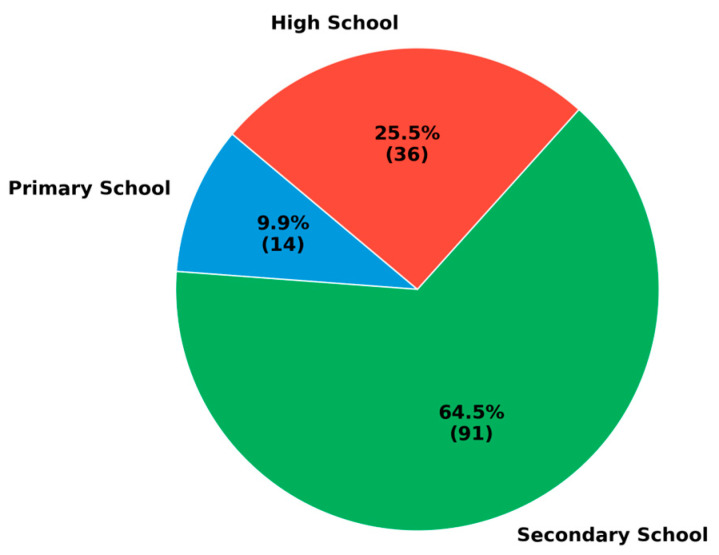
Distribution of teachers by school type (number and percentage).

A total of 141 teachers participated in this study; 89 of them were female and 52 were male. The participants were teachers living in Turkey and working in public schools. The distribution of participants according to regions is presented in Figure 1; the distribution of teachers according to branch categories is presented in Figure 2.

In this study, a comprehensive dataset was used in order to reach a wide range of teachers and to obtain the most realistic data. A large number of teachers were reached through the interview form prepared over the internet. This method increased the efficiency of the data collection process and reinforced the reliability of the research.

To better understand the sample, the school types in which the teachers work were also identified. As presented in Figure 2, the majority of the participants work in secondary schools, followed by those in high schools and primary schools.

**Figure 3:**
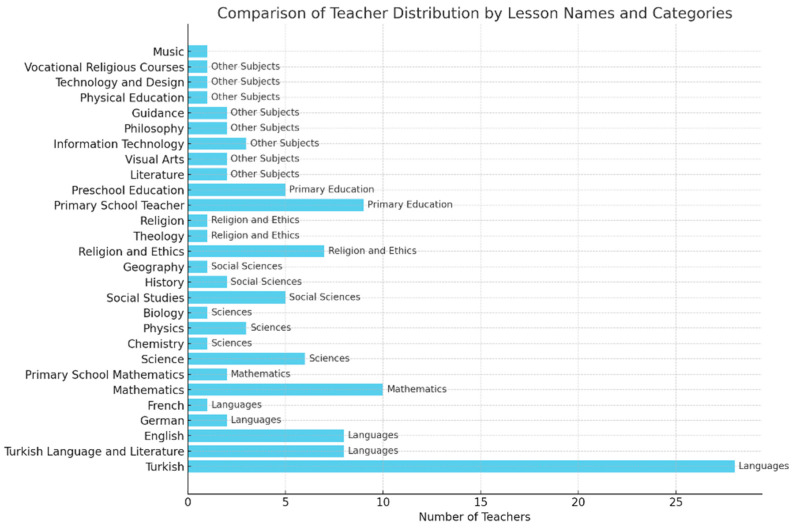
Comparison of Teacher Distribution by Lesson Names and Categories.

In addition to school types, teachers came from different branches, which were categorized into specific subject areas. This classification provides further insight into the professional backgrounds of the participants. The distribution of teachers by branch is shown in Figure 3.

In this study, the validity and reliability of the data were ensured through multiple methods. Data collection started with teachers already acquainted with the researcher and gradually expanded to include a broader group of participants through snowball sampling, using Google Forms. To enhance validity, demographic information such as teaching experience, gender, and educational levels was collected to ensure representativeness. Reliability was ensured through the standardization of interview procedures, wherein all participants were asked identical questions and the interviews were conducted following a consistent format. Member checking was also applied, with summaries of participants’ responses sent back for verification.

This systematic approach increased the validity and reliability of the study by providing detailed insights into teachers’ demographic distributions and their subject specializations.

### 2.3. Data Collection

In this study, the interview method, a qualitative research technique, was employed, and a semi-structured interview form was used as the primary data collection tool. To ensure the collection of in-depth and meaningful data, purposive sampling was applied, targeting experienced public-school teachers who had previously encountered ethical dilemmas in their professional practice. The interview form was carefully developed and refined based on feedback from five academic experts specializing in educational ethics and qualitative research.

As part of the data collection process, a series of ethical dilemma scenarios were designed to guide both teacher responses and AI interactions. The development of these scenarios was grounded in the framework proposed by Shapira-Lishchinsky (2011), which identifies five common categories of ethical dilemmas frequently experienced by teachers in educational contexts. This framework ensured that each scenario reflected realistic and profession-specific challenges likely to be encountered in daily teaching practice. To enhance the contextual relevance of the dilemmas, a comprehensive review of the literature was conducted to identify the ethical challenges most commonly faced by teachers (Aultman et al., 2009; Husu & Tirri, 2001; Klaassen, 2002; Lyons, 1990; Nakar, 2017; Wang et al., 2022). Particular attention was paid to ensuring that the scenarios captured the real-life complexities and professional tensions that educators navigate. This careful design process strengthened the authenticity of the dilemmas and ensured their suitability for comparing human and AI ethical reasoning.

Teacher responses were collected via Google Forms, providing participants with the opportunity to reflect on each dilemma and explain how they would act in the given situations. The collected responses were then exported to Excel and subsequently transferred to MAXQDA for systematic coding and qualitative analysis. MAXQDA facilitated the identification of recurring themes, reasoning patterns, and ethical perspectives within the teacher data.

To align with the teacher data collection process, an identical set of ethical dilemmas was also posed to various large language models (LLMs) to assess their capability in addressing complex ethical situations. Models reviewed during this preliminary stage included Gemini, Claude, Grok, and Copilot. Each model demonstrated strengths in general conversational abilities and scenario-based interactions. However, ChatGPT (GPT-4o) was ultimately selected due to its ability to generate coherent, contextually appropriate, and well-justified responses with a high level of consistency across scenarios. The model’s capacity to articulate reasoning and provide clear justifications aligned closely with the analytical requirements of the study. Additionally, ChatGPT’s widespread accessibility and established use in educational and research contexts further supported its selection as the most suitable model for this research.

To ensure the reliability of the AI responses and eliminate potential biases, a new ChatGPT account with no prior usage history was created specifically for the study. The ethical dilemmas were presented in fresh, unstructured chat sessions to prevent any influence from previous interactions or pre-existing data. During the AI data collection process, it was observed that ChatGPT initially provided multiple options rather than committing to a single response. To obtain definitive answers, a follow-up question—“Which of these options would you choose?”—was introduced. This prompt encouraged the model to select and justify a specific choice, allowing for a clearer and more direct comparison with human responses.

All AI-generated responses were compiled and transferred to MAXQDA for systematic coding and analysis, following the same procedure used for the teacher data. This structured approach ensured that both sets of data were analyzed consistently, enabling a comprehensive comparison of ethical decision-making processes between human educators and the AI system.

### 2.4. Data Analysis

The data were analyzed through content analysis and cross-case synthesis techniques, which are established qualitative research designs. Cross-case synthesis identifies similarities and differences by comparing multiple situations, thereby establishing connections among these aspects (Yin, 2017). According to Yin (2017), cross-case synthesis is applicable exclusively in multiple case analyses since it relates to various cases.

#### 2.4.1. Core Features

##### Data Entry and Management

MAXQDA supports various data formats, allowing users to efficiently import and manage qualitative data. Users can upload files directly or integrate external data sources into the software environment. In this study, interview forms collected from teachers were transferred into the MAXQDA program by the researchers.

##### Coding Functionality

One of MAXQDA’s core strengths is its coding functionality, which enables users to assign codes to text or other data segments, facilitating theme identification and data structuring for analysis. In this study, two researchers analyzed four interview forms and identified key ethical approaches as coding categories. These codes included Deontological Ethics, Virtue Ethics, Utilitarianism, Situational Ethics, and Social Justice Ethics.

The selection of these codes was guided by their prevalence in the interview forms. Deontological Ethics, Virtue Ethics, and Utilitarianism, being foundational ethical frameworks, were distinctly represented. Social Justice Ethics was included due to its frequent mention in teachers’ responses during the preliminary analysis. Although deontology and social justice ethics share some similarities, they differ significantly: deontology emphasizes rule adherence, while social justice ethics focuses on equality and rights. Situational Ethics was applied when teachers’ responses demonstrated context-based evaluations of events. This process provided insights into how teachers approached ethical dilemmas and revealed dominant ethical perspectives.

##### Content Analysis

MAXQDA facilitates systematic content analysis through coding, enabling the identification of key themes and patterns in the data. In this study, ethical approaches were defined as codes and teachers’ responses were analyzed accordingly. This method allowed for exploring relationships between codes and gaining insights based on the frequency and context of the coded data. In the third stage of analysis, one researcher applied the identified codes to the teachers’ responses and imported AI-generated responses (from ChatGPT) into the program. These responses were then analyzed using the same coding framework.

##### Querying and Analysis

MAXQDA offers robust querying tools that allow researchers to perform advanced analyses across datasets. Users can explore relationships between codes, examine code prevalence, and conduct detailed investigations. In the final stage, both researchers collaboratively reviewed the coding, resolved any errors, and generated cross-case tables using the program. These tables enabled a comparative analysis of teacher and AI responses. The cross-case synthesis revealed both similarities and differences in the ethical approaches of teachers and AI, providing a detailed understanding of their respective ethical perspectives.

##### SUM Calculation and Coding Process

The “SUM” values in the tables represent the total percentage of responses coded under each ethical dilemma category. However, certain questions elicited either no responses or invalid answers from teachers, leading to the exclusion of those items from the SUM calculation.

Consequently, the totals in certain categories may be less than 100%. Coding was performed only for valid and appropriate responses, ensuring the reliability of the data.

##### Visualization

MAXQDA offers various visualization tools to comprehensively represent analytical outcomes. The software includes options for generating visual representations such as concept maps, charts, and graphs, thereby enhancing the interpretability of the findings.

##### Reporting Capabilities

After completing analyses, users can seamlessly compile results into coherent reports. MAXQDA’s reporting module facilitates the organization and presentation of findings, making it convenient to share insights with stakeholders. The results are depicted using visualization tools, and comprehensive reports are generated to effectively communicate the findings.

## 3. Results

The responses of teachers and AI to the ethical dilemma scenarios were analyzed in detail in this study. The results derived for each ethical dilemma category, along with the differences between teachers and AI, are comprehensively presented in Table 2. The full list of ethical dilemma questions utilized in the study is provided in Appendix A.

Question 1. What is the relationship between the decision-making processes given by teachers and artificial intelligence in ethical dilemma scenarios?

### 3.1. The Dilemma of Moral Integrity and Social Responsibility

In this ethical dilemma, both the majority of teachers and the AI predominantly adopted a deontological ethical approach. A total of 48.2% of participants, along with the AI, based their decisions on the principles of honesty and justice, arguing that Mr. Bülent should report the incident to the school administration. The AI articulated this reasoning as follows: *“By adopting a deontological ethical approach, I believe Mr. Bülent should act by the principles of honesty and justice and inform the school administration of the truth. Explaining that Hasan accidentally caused the damage would help Mr. Bülent maintain his credibility as a teacher and act in line with the school’s principles of discipline and justice. Although the financial difficulties Hasan and his family will face are unfortunate, telling the truth and ensuring justice takes precedence in terms of social and professional norms”*.

Similarly, teachers who leaned toward deontological reasoning emphasized the importance of honesty both on an individual and institutional level. Representing this perspective, one participant stated: *“The situation should be reported to the administration. It should be communicated that the student is facing financial hardship, and this context ought to be taken into account. However, despite his difficult circumstances, there should still be appropriate consequences for the damage caused. This would send a clear message to both the student and his classmates about the importance of being more cautious”* (*P1*). Teachers sharing this view argued that telling the truth is morally necessary and that the incident could also serve as an educational opportunity for students. However, they also indicated that factors like the student’s financial status could be taken into account by the administration, showing that their stance was not entirely rigid.

Meanwhile, 28.4% of the teachers reflected a virtue ethics perspective, emphasizing the moral and humane aspects of the situation. Teachers with this viewpoint acknowledged the importance of justice but also expressed their willingness to personally take responsibility to prevent harm to the student. For instance, one participant suggested: *“He should report it to the administration but offer to pay for it himself.”* (*P8*). This response indicates a tendency among these teachers to prioritize empathy and conscience, seeking solutions that protect the student from harm.

The utilitarian approach appeared in 13.5% of responses, with participants focusing on minimizing harm, especially considering the student’s financial situation. A participant reflecting this view stated: *“Mr. Bülent should act as if he doesn’t know who caused the damage because the student’s financial situation is not good. Besides, it was an accident.”* (*P55*). Teachers with this perspective considered the accidental nature of the incident and the student’s hardship, arguing that concealing the truth might be a more humane choice in this particular context.

Overall, the responses revealed a variety of ethical perspectives among teachers, though a significant portion still prioritized honesty and justice. At the same time, a notable group of teachers displayed a contextual and solution-oriented mindset. Those who leaned toward virtue ethics emphasized moral responsibility and empathy, while utilitarian thinkers prioritized minimizing harm to the student.

Notably, just 19.1% of the participants reported facing a similar situation in their professional experience. This low percentage suggests that such institutional justice dilemmas are not very common in daily educational practice but can be quite challenging when they arise.

In general, while the AI displayed a more rule-based and principle-oriented approach, teachers tended to adopt responses that considered the context and human factors. However, it would be inaccurate to draw sharp distinctions between them. Some teachers who leaned toward deontological reasoning still acknowledged the need to consider the student’s circumstances, while the AI also described Hasan’s potential hardships as “unfortunate”. This suggests that there are overlapping points between the two perspectives.

Ultimately, these findings demonstrate that in ethical dilemmas within educational settings, teachers are capable of producing various solutions driven by conscience, empathy, and a desire to protect the student, while the AI remains largely within a systematic and principle-based framework. Nevertheless, the fact that the AI also recognized the student’s hardships points to the potential for such systems to approach human values to some extent.

### 3.2. The Dilemma of Justice and Cultural Sensitivity

In this ethical dilemma, a significant number of teachers adopted a social justice ethics perspective, emphasizing that cultural differences should not lead to unfair treatment within the classroom. Specifically, 48.9% of participants focused on ensuring equality and fairness, regardless of students’ backgrounds. For example, one teacher stated: *“In my opinion, she should implement the required disciplinary actions, keeping in mind that every student deserves equal treatment, whether local or foreign.”* (P57). Teachers who shared this view emphasized neutrality and fairness in decision-making but generally did not offer detailed strategies aimed at strengthening social cohesion in the classroom.

Additionally, several teachers approached the situation from a deontological ethics perspective, prioritizing duties and rules over context-specific factors. One participant noted: *“Ms. Selma should evaluate Ahmed’s situation impartially and inform the school administration.”* (P52). This response reflects a focus on procedural responsibility rather than emotional or cultural sensitivity.

Some teachers, however, leaned toward virtue ethics, suggesting more human-centered and empathetic solutions. These teachers emphasized that making mistakes is part of being human and that forgiveness is an essential part of education. For instance, one participant shared: *“It is truly a difficult situation. Ms. Selma can handle it by acknowledging that Ahmed did not act intentionally, that people make mistakes, and that everyone deserves to be forgiven at least once.”* (P35). This approach prioritizes understanding and forgiveness, considering the student’s intention and personal development.

Overall, while a large portion of the teachers emphasized social justice and equality, their approaches varied in terms of solutions. Some focused strictly on rules, while others leaned toward empathy and more flexible resolutions. Teachers advocating social justice prioritized fairness but often provided limited suggestions for enhancing social cohesion or addressing cultural sensitivities in practice.

At this point, it is noteworthy that the AI’s approach aligned with the majority of teachers who adopted a social justice perspective. Like many teachers, the AI recognized the importance of ensuring equality and fairness. However, compared to some teachers, the AI offered a broader perspective in its proposed solution. The AI’s response was: *“Ms. Selma should protect Cem’s rights by considering his victimization and questioning Ahmed’s actions to ensure justice and equality in the classroom. At the same time, she should foster empathy and understanding to prevent Ahmed and other foreign students from being excluded, and organize activities to promote tolerance and communication among students.”* (Artificial Intelligence).

In contrast to certain teacher responses, the AI’s recommendation not only focused on justice but also suggested concrete steps to strengthen social integration within the classroom. This indicates that the AI approached the dilemma from a broader perspective, balancing individual rights with classroom harmony.

According to the data, 46.1% of teachers reported encountering a similar ethical dilemma in their professional experience, while 53.9% stated they had not. This suggests that while cultural sensitivity dilemmas are not everyday occurrences, they arise often enough to require careful ethical consideration.

In conclusion, this scenario shows that the AI and the majority of teachers shared common ground in emphasizing social justice. However, the AI proposed a more comprehensive solution compared to some teachers, offering strategies aimed at fostering social harmony. Teachers, on the other hand, generally based their reasoning on equality and fairness but produced diverse ethical judgments depending on their experiences and values.

### 3.3. Equality and Management of Individual Differences

In this ethical dilemma, a clear divergence emerged between teachers and the AI. While 47.5% of the participants approached the situation from a social justice ethics perspective, the AI adopted a virtue ethics approach. Teachers who embraced the social justice perspective predominantly argued that all students should be treated equally and that academic success should not influence disciplinary decisions. One teacher stated, “The punishment should be consistent for all individuals involved; applying different consequences would constitute an injustice. Although Mustafa may excel academically, he is also expected to demonstrate the same level of competence in respect and proper conduct.” (P24). This perspective emphasizes that disciplinary measures should remain independent of students’ academic achievements, asserting that fairness necessitates uniform application of rules.

On the other hand, some teachers who adopted a virtue ethics perspective suggested that students’ overall character and behavior should be considered. One teacher remarked, “Administering identical punishments does not necessarily constitute justice. It is acceptable for Mustafa to receive different consequence, as considerations such as good behavior may warrant a reduced penalty”. This approach implies that Mustafa’s generally positive behavior could justify a lighter punishment.

Teachers with a utilitarian perspective focused more on the consequences of the behavior rather than past achievements. As one teacher explained, “The behavior should not be compared with academic success. The decision should be made based on the outcome of the behavior.” (P104), emphasizing that the disciplinary decision should be guided by the impact of the action rather than the student’s academic record.

The AI approached the dilemma through a virtue ethics lens but also placed significant emphasis on justice and appropriate sanctions. Although the AI acknowledged that Mustafa is generally a respectful and exemplary student, it stressed that his current behavior is unacceptable and should result in a proper punishment: “Mustafa’s offensive and provocative behavior is unacceptable and should be properly sanctioned. Similarly, if Mehmet also behaved inappropriately during the fight, he should receive an appropriate punishment.” (Artificial Intelligence, P3).

The AI suggested a solution where both students’ actions are assessed fairly within the context and appropriate disciplinary measures are applied. While the AI’s response included attention to character development, it prioritized ensuring justice and addressing the consequences of the behavior.

In this regard, the AI diverged from the majority of teachers by advocating for an evaluation that considers students’ overall character while also clearly emphasizing the need for fair and appropriate sanctions. The AI’s approach shows that despite choosing virtue ethics, it maintained a balance between justice, discipline, and attention to individual traits, rather than purely focusing on a human-centered or lenient perspective.

According to the data, 49.7% of teachers reported encountering a similar dilemma, while 50.3% stated they had not. This indicates that such dilemmas involving individual differences and discipline arise occasionally but are not universally experienced.

In conclusion, the majority of teachers in this scenario leaned toward a social justice approach, advocating equality in disciplinary decisions. The AI, however, diverged from this general trend by adopting a virtue ethics perspective and attempting to balance justice, appropriate sanctions, and consideration of individual character. The AI’s solution differed from some teachers’ strict equality stance by suggesting that justice may require evaluating the situation and student characteristics together.

### 3.4. The Dilemma of Individual Needs and Collective Responsibility

In this scenario, teachers and AI displayed distinct ethical approaches to handling the dilemma. While the AI adopted a utilitarian perspective, focusing on overall benefit and outcomes, the majority of teachers (53.9%) preferred virtue ethics, emphasizing fairness, empathy, and moral values. Teachers who leaned toward virtue ethics considered the student’s background and intentions, suggesting balanced responses that protected the individual while still acknowledging the behavior. One teacher stated, *“Gizem should receive a mild disciplinary response; however, the teacher should also offer her protection, recognizing that her behavior is unintentional and that she has unique needs or characteristics.”* (P69), reflecting an understanding of the student’s situation and the need for measured discipline.

A number of teachers opted for a deontological approach, prioritizing responsibility and institutional duty. One teacher straightforwardly recommended *“referring Gizem to the school administration.”* (P58), indicating that regardless of the student’s situation, the process should be carried out according to the rules.

Another group of teachers adopted a utilitarian perspective similar to the AI. One teacher explained, *“She should talk to Gizem, explain that her life situation negatively affects her behavior, and provide support. Meanwhile, she should seek the administration’s support. If the behavior continues, action should be taken considering the other students.”* (P81). This view aims to balance the needs of the individual and the classroom, offering initial support but protecting the class environment if necessary.

The AI, consistent with a utilitarian approach, proposed a solution that considered both the individual student and the collective benefit of the class. In its shortened version, the AI stated: *“In this case, I would adopt a utilitarian approach. (…) The teacher should consider Gizem’s difficult family situation and the underlying reasons for her behavior and seek alternative solutions rather than immediately reporting to the administration. (…) Collaborating with the guidance counselor and providing psychological support could help address the root causes. (…) If these efforts fail, reporting the case to the administration may be necessary as a last resort.”* (Artificial Intelligence, P4).

The AI’s response reflects a gradual process focused on supporting the student while also considering the classroom’s needs. It emphasizes first trying supportive measures but leaves room for administrative action if required. The AI’s approach aims to maximize benefits while minimizing harm, considering both Gizem’s future and the classroom environment.

The data show that 75.2% of teachers had encountered similar dilemmas in their careers, indicating that ethical challenges involving student behavior, assessment, and individual circumstances are common in educational settings.

The majority of teachers approached this dilemma through virtue ethics, offering solutions based on empathy and protecting the student’s dignity. Teachers who took a deontological stance emphasized duty and procedural adherence. Some teachers adopted a utilitarian perspective similar to the AI, attempting to balance individual support with classroom needs. The AI, however, proposed a structured solution focused on minimizing harm and maximizing benefit, addressing both individual needs and collective well-being. This contrast shows that while most teachers relied on moral values, the AI handled the situation in a systematic, outcome-focused manner.

### 3.5. The Dilemma of Fair Assessment and Rewarding Student Effort

This ethical dilemma revealed a diverse range of responses among teachers, reflecting the complexity of balancing individual student needs with institutional fairness. While the AI strictly adopted a deontological stance, only 45.4% of teachers aligned with this approach. A significant portion preferred virtue ethics (18.4%) or utilitarian reasoning (15.6%), indicating that many educators are willing to apply more flexible and context-driven ethical judgments.

Teachers who followed a deontological approach emphasized adherence to rules and objectivity in their assessment. One teacher explained, *“Unfortunately, although our education system tries to operate within a constructivist framework, the behaviorist grading mentality still prevails. In this system, we are compelled to grade the student according to their actual exam performance. Therefore, the teacher should give the score based on the exam paper.”* (P7). This response reflects a sense of professional obligation and the pressure of institutional expectations in grading practices.

On the other hand, teachers adopting virtue ethics focused on empathy, fairness, and protecting the student’s morale. One teacher shared, *“The teacher shouldn’t risk losing İrem. Exams are merely assessment tools—they have limitations and can’t capture the full picture. For this reason, the teacher should take initiative and consider increasing her grade.”* (P88). This perspective highlights the belief that rigid grading does not always reflect the student’s effort and overall learning and that moral responsibility sometimes requires going beyond the strict application of rules.

The AI, in contrast, maintained a deontological position, focusing on fairness, objectivity, and rule adherence. The AI suggested: *“In this case, I would adopt a deontological ethics approach. (…) The teacher should grade the exam as it is to ensure fairness and objectivity, treating all students equally. While it is important to recognize İrem’s efforts, changing the grade could create a perception of unfairness. Instead, the teacher can acknowledge her efforts in other ways, such as giving her leadership roles in-class activities or awarding a certificate of appreciation.”* (Artificial Intelligence, P5).

The AI’s response reveals a careful balance—protecting the integrity of the assessment while proposing alternative ways to support the student without compromising fairness. Although its primary commitment remains to objective grading, the AI acknowledges the importance of student motivation and morale, suggesting solutions outside the grading system.

Data shows that 76.6% of teachers have faced similar dilemmas, suggesting that tensions between individual care and institutional fairness are common in education.

Teachers’ responses demonstrate the ethical struggle between following rigid assessment rules and recognizing student effort and emotional needs. While some teachers remained committed to procedural fairness, others sought ways to reward effort, even at the risk of bending the rules. The AI, on the other hand, maintained strict fairness in grading but recommended complementary methods to support the student. This difference reflects how human educators may prioritize empathy in specific contexts, whereas the AI consistently applies principles of fairness and objectivity.

### 3.6. The Dilemma of Confidentiality and Professional Help

In this dilemma, both teachers and artificial intelligence predominantly adopted a virtue ethics approach, emphasizing empathy, respect for student dignity, and careful ethical judgment when addressing sensitive student situations. With 67.4% of teachers choosing virtue ethics, this reflects their strong emphasis on fairness, understanding, and prioritizing student well-being.

Teachers who embraced virtue ethics highlighted open communication and emotional support. One teacher stated, *“I would first talk to Mert about this. I would tell him that I could help, but I couldn’t do it alone and that we needed support. I would try to convince and guide him.”* (P1). This response reflects a student-centered approach that values empathy and trust while involving the student in the process.

On the other hand, some teachers adopted a deontological approach, emphasizing legal and professional obligations. One teacher explained, *“This is a difficult situation. What must be done is to seek help from the necessary authorities because it is a legal obligation.”* (P103). This perspective prioritizes adherence to laws and procedures over concerns about student privacy.

The artificial intelligence also adopted virtue ethics and described the approach as follows: *“In this case, I would adopt virtue ethics. (…) The teacher should respect Mert’s privacy while considering his well-being. The teacher should first meet Mert privately to discuss the concerns raised in his essay. It would be important to explain how involving the school counselor might help him and seek his consent. If Mert agrees, the process can begin; if not, the teacher should continue offering support and gradually try to encourage him to seek help.”* (Artificial Intelligence, P6).

According to the data, 76.6% of teachers had encountered similar dilemmas in their professional careers. This high rate indicates that managing individual differences while maintaining ethical boundaries is a common and challenging issue in educational settings.

Teacher responses in this scenario reflect the ethical tension between legal or institutional duties and the moral responsibility to protect and support the student. While some teachers adhered strictly to procedural correctness, the majority aligned with the AI’s virtue ethics approach, valuing empathy, patience, and respect for student autonomy. The AI reinforced this direction by offering a structured and thoughtful path that preserved both ethical principles and the student’s best interests.

### 3.7. The Dilemma of Measurement and Evaluation Ethics

In this dilemma, both the artificial intelligence and the majority of teachers favored a deontological approach, prioritizing fixed rules and moral principles when addressing issues of fairness and cultural sensitivity. With 60.3% of teachers choosing deontological ethics, this indicates that both AI and human educators value objectivity, honesty, and adherence to principles in such situations.

Teachers who adopted deontological ethics emphasized the importance of accepting the exam results as they were. One teacher explained, *“Ali should accept the exam results as they are. However, if the school administration insists, he should request a written document. The administration will likely not provide such a document, and the matter will be resolved.”* (P32). This response reflects the teacher’s effort to maintain professional integrity while managing potential administrative pressure.

On the other hand, some teachers preferred a utilitarian approach, focusing on learning outcomes and long-term benefits. One teacher stated, *“The exam should be repeated. If the majority fails, there is a problem with learning. A similar-level exam can be prepared and repeated to prevent learning errors. This is not to please parents but to fix learning problems.”* (P57). This perspective aims to support student learning and address potential gaps in understanding.

The artificial intelligence also adopted a deontological stance, stating: *“In this case, I would adopt a deontological ethics approach. (…) If Ali believes that the exam accurately measures the students’ real level, he should accept the results. Repeating or making the exam easier could send the wrong message and prevent students from recognizing their shortcomings. The right and fair action is to accept the results reflecting their actual level. Ali can explain the purpose and importance of the exam to parents and the school administration.”* (Artificial Intelligence, P7).

According to the data, 61.7% of teachers had faced similar dilemmas in their professional careers. This suggests that issues involving fairness, parental expectations, and academic integrity frequently arise in educational contexts.

Overall, teacher responses were divided: while one group strongly advocated for rules and fairness, the other focused on improving the learning process. In this dilemma, the artificial intelligence aligned with the majority of teachers, demonstrating a shared stance. This indicates that both AI and educators tend to make principle-based decisions when addressing cultural and justice-related challenges in educational settings.

### 3.8. The Dilemma of Individual Needs and Institutional Justice

In this dilemma, the artificial intelligence preferred the situation ethics approach, making ethical decisions based on contextual flexibility and empathy rather than rigid principles. In contrast, the majority of teachers (41.1%) adopted virtue ethics, shaping their decisions around values such as compassion, fairness, and integrity.

Teachers who adopted virtue ethics emphasized the importance of protecting the student from being harmed. One teacher emotionally stated, *“Oh, Kamil! I would never allow him to be victimized. I would ask the teacher to repeat the exam for Kamil.”* (P3). This response centers on protecting the student and reflects a stance where personal ethical values outweigh institutional expectations.

On the other hand, a teacher who preferred a utilitarian approach argued that flexibility could be applied as long as the individual need does not negatively impact other students: *“Laws and rules are valuable only as long as they serve people. Therefore, since passing Kamil would not affect other students, I would do everything I could to help him pass. (The reasons for his failure are very important here)”* (P103). This perspective highlights the importance of weighing potential harm and benefit, suggesting that rules can be flexibly applied in certain situations.

The artificial intelligence also adopted a situational ethics approach, attempting to balance empathy and fairness. The AI explained: *“In this case, I would adopt a situation ethics approach. (…) Emine should inform the school administration of Kamil’s family issue and request an exception to the exam policy. This would protect Kamil’s future and ensure he does not lose his chance to attend the high school he dreams of. At the same time, Emine could propose developing a policy for such cases to maintain fairness for all students.”* (Artificial Intelligence, P8).

The AI’s response demonstrates an effort to protect the student’s individual needs while also considering institutional justice. It also emphasizes the importance of creating a general policy for similar cases in the future.

According to the data, only 38.3% of teachers reported encountering a similar dilemma, while 61.7% had not. This indicates that although moral integrity and social responsibility are important in education, such ethical issues may not frequently arise or be explicitly addressed in daily teaching practices.

Overall, teachers’ responses reflect a strong emphasis on ethical principles and empathy. In contrast, the AI tended to focus on achieving a balance between fairness and adaptability by relying on contextual analysis. This contrast highlights that while AI leans toward procedural solutions, teachers tend to act based on ethical values and the unique needs of the student.

## 4. Discussion

Ethical decision-making is a cornerstone of effective teacher education, as educators consistently face complex moral dilemmas in the diverse and dynamic classroom environments. Previous studies have mainly focused on the ethical dilemmas faced by teachers and how they resolve these dilemmas (Aultman et al., 2009; Campbell, 2006; Ehrich et al., 2011; Buzzelli & Johnston, 2001; Colnerud, 2006; Husu & Tirri, 2001; Klaassen, 2002; Rice, 2001; Shapira-Lishchinsky, 2011; Wang et al., 2022). However, this study compares the ethical decision-making processes of teachers with the responses generated by artificial intelligence (AI), analyzing the similarities and differences between the two approaches.

In this context, we examined the responses of teachers and AI to ethical dilemmas and analyzed how different ethical approaches emerged. First, we identified the key ethical dilemmas that teachers frequently encounter and then compared their responses with those generated by AI. While developing the ethical dilemma scenarios, we based our study on the five main categories of ethical dilemmas identified by Shapira-Lishchinsky (2011): the conflict between a caring and formal school climate, balancing distributive justice with school standards, the contradiction between confidentiality principles and school rules, the tension between loyalty to colleagues and school norms, and the conflict between family expectations and educational standards. These dilemmas were presented as structured scenarios, allowing us to analyze the ethical decision-making patterns in detail.

Our findings indicate that in five ethical dilemmas, teachers and AI reached similar decisions, while significant differences emerged in three cases. In the dilemmas titled “Moral Integrity and Social Responsibility”, “Justice and Cultural Sensitivity”, “Fair Assessment and Rewarding Effort”, “Confidentiality and Professional Help”, and “Assessment and Evaluation Ethics”, both teachers and AI predominantly adopted structured ethical approaches such as deontological ethics and social justice ethics. Notably, in the “Confidentiality and Professional Help” dilemma, both sides emphasized virtue ethics. This alignment can be seen as a reflection of long-standing professional ethical standards and value-oriented approaches embraced in the field of education.

On the other hand, significant differences emerged in the dilemmas of “Managing Individual Differences”, “Balancing Individual Needs with Collective Responsibility”, and “Balancing Individual Needs with Institutional Justice”. In these cases, AI exhibited utilitarian or situational ethical approaches, while teachers balanced their decisions among virtue ethics, social justice, and deontological ethics. This difference demonstrates that teachers consider contextual factors, pedagogical experience, and the human element more deeply in their ethical decision-making processes.

AI, however, operates primarily through a results-oriented mechanism based on predefined rules. Yet, this analytical approach does not imply that AI’s decisions are entirely objective or impartial. AI systems largely rely on the datasets on which they are trained, and these datasets may contain cultural assumptions, implicit biases, or incomplete representations. Such factors directly influence AI’s ethical preferences. Especially in a value-laden field like education, the content and quality of the data used shape the ethical approach that AI adopts. Therefore, AI’s adoption of utilitarian or situational ethics in certain dilemmas cannot be explained solely by analytical calculations; the limitations and inherent biases of the data sources also play a significant role in these decisions.

For this reason, the ethical decisions made by AI in a complex, human-centered field such as education should be evaluated not only based on outcome-oriented algorithms but also by considering the quality, diversity, and potential biases of the data sources.

Our analysis further revealed that teachers’ ethical decision-making processes are influenced by personal experiences, emotions, and professional responsibilities. Teachers often struggle to balance individual student needs with institutional expectations and, in some cases, prioritize student well-being over strict adherence to rules. For example, when a student causes damage, some teachers prefer to report the incident to the administration on the condition that the student pays for the damage, thus ensuring the student takes responsibility while the teacher makes certain sacrifices to manage the process in favor of the student. Similarly, when a student faces a special personal situation, some teachers tend to relax the rules and make decisions that prioritize the student’s well-being.

These findings are consistent with the existing literature and demonstrate that teachers tend to prioritize social justice and virtue ethics when making ethical decisions. Ehrich et al. (2011) found that teachers rely on empathy and human-centered values when dealing with ethical dilemmas. In this regard, teachers adopt strategies such as sharing their experiences with trusted colleagues, developing internal ethical frameworks to prevent harmful actions, and explicitly expressing their professional ethical principles.

Similarly, Shapira-Lishchinsky (2011) found that teachers often base their ethical decisions on incidents from the early stages of their careers and that their experiences with negative situations influence their decision-making processes. Our findings support the idea that teachers’ responses to ethical dilemmas are shaped by personal ethical principles, professional responsibilities, and contextual factors. In contrast, AI tends to adopt a more analytical approach, selecting the most effective or utilitarian option. This distinction highlights the fundamental differences between teachers and AI in ethical decision-making.

Teachers’ adoption of diverse and human-centered approaches allows them to engage with students more empathetically and equitably. Husu and Tirri (2001) argue that teachers prioritize “the best interest of the child” when resolving ethical dilemmas and that empathy plays a crucial role in this process. This perspective suggests that teachers not only navigate ethical challenges involving students but also mediate conflicts between colleagues and parents with an empathetic approach.

On the other hand, AI’s analytical and rule-based decision-making processes can serve as a useful guide for handling ethical dilemmas. Casas-Roma et al. (2021) discuss the role of AI in resolving ethical dilemmas and emphasize the importance of integrating “ethical sensors” into autonomous systems to enhance AI’s ethical awareness. Such tools could enable AI to more accurately analyze ethical scenarios and better assess contextual variables.

Our study focuses on real-life ethical dilemmas encountered by teachers throughout their careers. Liu (2023) suggests that the ability of advanced AI systems to synchronize cultural, social, and individual differences in decision-making can contribute to fostering harmony among diverse value systems. AI-generated responses are claimed to undergo comprehensive filtering, review, and analysis based on multiple parameters. However, our findings indicate that teachers’ ethical decisions are influenced by factors such as gender, years of professional experience, and the educational level at which they teach.

This study provides valuable insights into the differences between teachers’ ethical approaches and AI-generated responses. It underscores the importance of integrating such findings into educational policy-making and supporting ethical decision-making processes. Enhancing societal awareness and promoting more informed ethical decisions require a balanced evaluation of both human-driven and AI-driven approaches.

## 5. Conclusions

The growing integration of artificial intelligence (AI) into education has raised critical questions on the relationship between technological tools and human-centered decision-making. AI’s analytical and rule-based decision-making processes can offer consistent and predictable outcomes in ethical dilemmas. Therefore, it is crucial to consider the strengths of both approaches and strike a balance between them.

In educational settings, teachers frequently encounter complex ethical dilemmas that do not have clear-cut solutions. In such situations, every possible decision carries different short- and long-term consequences and impacts. Teachers may not always foresee all possible outcomes of their decisions under the given circumstances. Moreover, factors such as stress, time pressure, and emotional load can narrow their reasoning and judgment.

At this point, AI can serve as a guide or supportive tool for teachers. When an ethical dilemma is presented to AI, it can generate possible decision alternatives along with simulations or scenario-based projections of the potential short- and long-term consequences of each choice. This allows teachers to evaluate their decisions not only in terms of immediate outcomes but also in light of future implications, providing them with a broader perspective.

However, it is crucial to regard AI not as a “decision-maker” but as a “guiding tool” in this process. The suggestions or scenarios generated by AI should not be implemented without critical evaluation. Human experience, contextual knowledge, and moral reasoning must always remain in the hands of the teacher. AI should not be turned into an authority that surpasses human judgment and values. Instead, teachers should utilize AI to explore alternative perspectives, uncover potential outcomes they might have overlooked, and strengthen their reasoning skills. Such an approach can enrich teachers’ decision-making processes while simultaneously enhancing their professional ethical awareness.

In this context, incorporating AI-driven scenario analysis and simulations into teacher training programs can be highly effective. Pre-service teachers can engage with AI-supported ethical dilemma scenarios, allowing them to develop professional reasoning skills and practice evaluating diverse situations from multiple perspectives. Thus, AI can become not only a support tool for in-service teachers but also a vital component of teacher education.

AI has the potential to play a crucial role in promoting both a sustainable d bad and equitable decision-making processes. By leveraging vast datasets and sophisticated algorithms, AI can facilitate informed decision-making that takes into account not only immediate results but also long-term impacts on the environment and society. Moreover, integrating AI into decision-making processes can enhance fairness and equity in education and other fields. By employing data-driven approaches, AI systems can help eliminate biases that may exist in human decision-making, thereby supporting a more just allocation of resources and opportunities. In educational environments, AI can ensure the provision of personalized learning experiences to all students, regardless of their backgrounds or abilities, thus contributing to a more inclusive and equitable educational setting.

However, it is essential to recognize that ethical implementation of AI is critical to realizing these benefits. Ensuring transparency, accountability, and inclusivity in AI systems is vital to building trust and guaranteeing that AI-driven decisions align with principles of fairness and social justice. In conclusion, by combining the strengths of human-centered approaches with the analytical capabilities of AI, it is possible to create a more equitable world that upholds justice and benefits all members of society.

### Future Research and Applications

Future research can further explore teachers’ ethical decision-making processes and uncover additional factors influencing these processes. Moreover, more studies are needed that compare teachers’ ethical decision-making with AI-driven processes. Accordingly, cross-cultural research could provide insights into how values and norms shape ethical decisions globally. Such research can contribute to a better understanding and improvement of ethical decision-making in education. Additionally, studies can focus on the role of teacher education programs in fostering ethical competence. This may include evaluating the effectiveness of case-based learning, simulations, and AI-assisted ethical training modules in enhancing reflective thinking and moral sensitivity. Furthermore, longitudinal studies could examine how ethical reasoning evolves over time and across different teaching contexts. There is a growing need to investigate how pre-service and in-service teachers perceive, interpret, and respond to ethical dilemmas. Incorporating these findings into the development of educational policies and programs will help create more equitable, sustainable, empathetic, and effective educational environments.

## Figures and Tables

**Table 1:** Approaches to ethical dilemmas.

Ethical Approach	Defenders	Years	Definitions
Deontology	Immanuel Kant	18th Century	An ethical approach that argues that moral rules are universal and binding.
Utilitarianism	Jeremy Bentham, John Stuart Mill	18th and 19th Century	An ethical approach aimed at ensuring the greatest happiness as a result of actions.
Virtue Ethics	Aristotle	4th Century B.C.	An ethical approach that emphasizes virtuous behavior and character development.
Ethics of Social Justice	John Rawls	20th Century	An ethical approach that aims to protect the rights of the weakest individuals in society.
Situation Ethics	Joseph Fletcher	20th Century	A flexible ethical approach that argues that moral decisions can change according to the situation.

**Table 2:** Comparison of teachers’ and AI’s responses to ethical dilemma scenarios.

	Artificial Intelligence (AI)	Teachers	Total
**Moral Integrity and Social Responsibility Dilemma**			
Situational Ethics	0	5.7%	5.6%
Social Justice Ethics	0	2.8%	2.8%
Virtue Ethics	0	28.4%	28.2%
Deontological Ethics	100.0%	48.2%	48.6%
Utilitarianism	0	13.5%	13.4%
**Justice and Cultural Sensitivity Dilemma**			
Situational Ethics	0	2.8%	2.8%
Social Justice Ethics	100.0%	48.9%	49.3%
Virtue Ethics	0	31.9%	31.7%
Deontological Ethics	0	16.3%	16.2%
Utilitarianism	0	0	
**Equality and Managing Individual Differences**			
Situational Ethics	0	7.8%	7.7%
Social Justice Ethics	0	47.5%	47.5%
Virtue Ethics	100.0%	19.1%	19.7%
Deontological Ethics	0	24.1%	23.9%
Utilitarianism	0	1.4%	1.4%
**Individual Needs and Collective Responsibility Dilemma**			
Situational Ethics	0	14.2%	14.1%
Social Justice Ethics	0	4.3%	4.2%
Virtue Ethics	0	53.9%	53.5%
Deontological Ethics	0	27.0%	26.8%
Utilitarianism	100.0%	2.8%	3.5%
**Fair Assessment and Rewarding Effort Dilemma**			
Situational Ethics	0	7.1%	7.0%
Social Justice Ethics	0	12.8%	12.7%
Virtue Ethics	0	18.4%	18.3%
Deontological Ethics	100.0%	45.4%	45.8%
Utilitarianism	0	15.6%	15.5%
**Privacy and Professional Help Dilemma**			
Situational Ethics	0	2.8%	2.8%
Social Justice Ethics	0	0.7%	0.7%
Virtue Ethics	100.0%	67.4%	67.6%
Deontological Ethics	0	16.3%	16.2%
Utilitarianism	0	14.2%	14.1%
**Ethics of Assessment and Evaluation Dilemma**			
Situational Ethics	0	8.5%	8.5%
Social Justice Ethics	0	4.3%	4.2%
Virtue Ethics	0	3.5%	3.5%
Deontological Ethics	100.0%	60.3%	60.6%
Utilitarianism	0	23.4%	23.2%
**Individual Needs and Institutional Justice Dilemma**			
Situational Ethics	100.0%	10.6%	11.3%
Social Justice Ethics	0	12.8%	12.7%
Virtue Ethics	0	41.1%	40.8%
Deontological Ethics	0	12.8%	12.7%
Utilitarianism	0	19.9%	19.7%
SUM	800.00	797.87	797.89
N = Documents	100.00	100.00	100.00

Note: Since AI (ChatGPT) represents a single model, its responses are entirely directed toward a specific option (100%). In contrast, teacher responses are distributed based on sample size.

## Data Availability

The data supporting the findings of this study are available within the article.

## Appendix A

The Moral Integrity and Social Responsibility Dilemma: Mr. Bülent, an English teacher, discovers that the school’s smartboard has been seriously damaged. Upon conducting an investigation, he finds out that the student responsible is Hasan, who comes from a financially struggling family and caused the damage unintentionally. Aware that Hasan cannot cover the cost and that doing so would place a heavy burden on his family, Mr. Bülent faces a difficult situation. The school administration demands that the person at fault be identified, disciplined, and held financially accountable. However, Mr. Bülent is unable to help the family financially himself, and opposing the administration’s stance could jeopardize his professional standing and job security.

In this situation, Mr. Bülent faces two difficult choices: concealing the truth to consider Hasan’s situation or telling the administration the truth, causing financial harm to Hasan and his family. What decision do you think Mr. Bülent should make in this situation, and why?

Justice and Cultural Sensitivity Dilemma: Social studies teacher Ms. Selma is faced with a difficult situation following a fight between her foreign student Ahmed and Turkish student Cem, resulting in Cem sustaining minor injuries and significant tension in the class. Upon investigating, Ms. Selma learns that Ahmed had previously participated in a joke against Cem, which bothered him. Other students’ statements indicate that Cem is generally a calm and compliant student.

Students’ Preference: All students except a few foreign ones support Cem. They believe Cem is justified because of Ahmed’s previous behavior. The other foreign students remain silent and follow the events passively. The rest of the students focus on Cem’s victimization and think he is wrong.

In this situation, Ms. Selma needs to support Cem considering his victimization and question Ahmed’s actions based on her sense of justice. However, this decision could increase social pressure and lead to the exclusion of Ahmed and other foreign students. What do you think Ms. Selma should do in this situation?

Equality and Managing Individual Differences: Ms. Yasemin, a math teacher, witnesses a confrontation between her top-performing and favorite student, Mustafa, and Mehmet, who is typically disengaged and unmotivated. The altercation was triggered by Mustafa’s offensive remarks toward Mehmet, which led to the fight. Although Mustafa is generally respectful and serves as a model student, Ms. Yasemin considers his actions in this incident to be inappropriate and unacceptable.

In this situation, should Ms. Yasemin give Mustafa a different punishment than Mehmet? Do you think it would be fair if Ms. Yasemin gave Mustafa a different punishment? What decision would you make if you were in Ms. Yasemin’s place?

Individual Needs and Collective Responsibility Dilemma: A science teacher observes that a student named Gizem is repeatedly disrupting her lessons. Gizem’s actions interrupt the flow of the class and divert the attention of her classmates. Despite repeatedly warning Gizem, the behavior continues. The teacher also knows that Gizem has a difficult family life, which might be the underlying cause of her actions. Reporting this to the school administration would result in significant disciplinary actions for Gizem, possibly even expulsion. On the other hand, ignoring the situation negatively affects the education of other students.

In this situation, the teacher faces two difficult choices: not reporting the situation to the administration considering Gizem’s difficult family life or reporting it to maintain classroom order and risking significant disciplinary actions for Gizem. What do you think the teacher should do in this situation, and why?

Fair Assessment and Rewarding Effort Dilemma: A social studies teacher has a dedicated and actively participating student named İrem. İrem regularly attends classes, completes her assignments on time, and actively contributes to class discussions. However, she unexpectedly fails the year-end social studies exam. Now, the teacher must make a choice regarding İrem’s grade:

Fair Grading: The teacher can leave İrem’s exam grade as it is, ensuring fair assessment. This would be fair to other students but may not reward İrem’s efforts adequately.

Rewarding Effort: The teacher can raise İrem’s grade considering her class participation and efforts. This could boost İrem’s morale and reward her efforts but might create a perception of unfairness among other students and question the objectivity of grades. What do you think the teacher should do in this situation?

Privacy and Professional Help Dilemma: A Turkish teacher asks her students to write a composition on a personal topic. A student named Mert writes about a difficult period in his life, hinting at some harmful habits without directly mentioning them. The teacher realizes that Mert’s composition is quite sensitive and also observes that writing about this topic might have provided Mert with some emotional relief.

The teacher faces a dilemma: whether to share Mert’s situation with the school counselor. Sharing could provide Mert with professional help for his problems but might also violate his privacy and break his trust. On the other hand, not sharing the situation could miss the opportunity to intervene in Mert’s issues, potentially affecting his future negatively.

In this scenario, the teacher must choose between maintaining trust with the student and providing the necessary help. What do you think the teacher should do in this situation?

Ethics of Assessment and Evaluation Dilemma: Math teacher Mr. Ali conducts an exam for his class, and most of the class fails. The school administration asks Mr. Ali to retake the exam and prepare an easier one to improve the school’s overall success rate and alleviate parent’s reactions. However, Mr. Ali believes that the difficulty of the exam is necessary to measure the students’ actual levels and is concerned that retaking the exam might send the wrong message to the students. Mr. Ali has never faced such a situation in his teaching career and is uncertain about the best course of action.

In this situation, what decision do you think Mr. Ali should make? Should he allow a retake to satisfy the school’s and parents’ expectations, or should he stand by the original results to accurately represent the students’ actual performance levels? What do you think would be the best decision for Mr. Ali and why?

Individual Needs and Institutional Justice Dilemma: Turkish teacher Ms. Emine learns that Kamil failed the Turkish exam, resulting in him losing the right to take the LGS (High School Entrance Exam). Ms. Emine knows that Kamil is normally a successful student and had a significant family issue on the exam day. However, the school’s exam policy is very strict and does not allow any exceptions. Additionally, if Kamil cannot take the LGS, he will lose the chance to study at his dream high school. On the other hand, Ms. Emine’s professional reputation and adherence to the school’s fairness principle are also at stake.

In this scenario, Ms. Emine must make a very difficult decision between protecting Kamil’s future and adhering to the school’s fairness principle and her professional reputation. What do you think Ms. Emine should do in this situation?

## Appendix B

The initial conversation with ChatGPT (GPT-4o) was conducted in Turkish to align with the context and language of the study. To preserve the originality and natural flow of the interaction, the full conversation was not re-generated in English. Instead, a summary of the English transcript is provided below to represent the AI’s reasoning and responses.

The original AI interaction can be accessed via the following link: https://chatgpt.com/share/67dbfbea-c3a8-800c-9334-8d4348a36307 (accessed on 22 March 2025).

This summary reflects the model’s ethical reasoning and decision-making patterns as demonstrated in the original conversation. While the wording may differ in English, the core reasoning remains consistent with the AI’s initial responses.

### English Transcript Sum

**User:** I’d like you to respond to some ethical dilemma scenarios I’ll present.

**ChatGPT:** Of course! Send me the scenarios, and I’ll be happy to help you answer each one.

**User:** 1. Bülent, an English teacher, notices that the school’s smart board has been seriously damaged. After looking into the matter, he discovers that one of his students, Hasan—who comes from a financially disadvantaged background—accidentally caused the damage. Bülent is aware that Hasan’s family cannot afford to cover the cost, and doing so would place them under significant financial strain. However, the school administration insists that whoever is responsible must be identified, disciplined, and required to pay for the damage. Bülent is not in a position to help financially, and going against the administration could harm his professional standing and possibly his job security. Now, he faces a difficult decision: should he conceal Hasan’s involvement to protect the student’s family, or report it to the administration, knowing it will financially hurt them? What should Bülent do, and why?

**ChatGPT:** In such a dilemma, Bülent faces two core values: honesty and justice vs. compassion and empathy. Analyzing through different ethical lenses:

Deontological Ethics: He must tell the truth because being honest is a universal moral rule. Despite the consequences, truth and justice are fundamental.

Consequentialism (Utilitarianism): Hiding the truth could prevent Hasan’s family from suffering, but honesty preserves the school’s discipline and fairness.

Virtue Ethics: Bülent should be both honest and compassionate. He could talk to Hasan’s family, and then approach the administration seeking alternatives like a school fund. Conclusion: Bülent should find a balanced solution—acknowledge Hasan’s situation but still inform the school while advocating for solutions that reduce harm to Hasan’s family.

**User:** If you had to choose one approach, what would it be? Which one is more reasonable?

**ChatGPT:** I would choose Deontological Ethics. Telling the truth is morally obligatory. While Hasan’s family’s situation is sad, honesty and justice should prevail for the sake of long-term trust, discipline, and fairness.

**User:** Can you write that in a single paragraph?

**ChatGPT:** Adopting a deontological approach, I believe Bülent should uphold honesty and justice by telling the school administration the truth about Hasan’s involvement. This protects his integrity as a teacher and aligns with the school’s disciplinary principles. Although Hasan’s family may face hardship, maintaining honesty ensures trust, fairness, and sets an important example of accountability for all students.

**User:** 2. Selma, a social studies teacher, is confronted with a difficult situation following a conflict between Ahmed, a foreign student, and Cem, a local student who ends up injured. Most students support Cem, and there’s a history of Ahmed bullying him. While the local students are vocal in defending Cem, the foreign students remain silent. Selma is aware that openly siding with Cem might escalate social tensions and further isolate Ahmed along with the other foreign students. Faced with this dilemma, what course of action should Selma take, and why?

**ChatGPT:** I would choose Social Justice Ethics. Selma should ensure fairness and equality by acknowledging Cem’s victimhood and holding Ahmed accountable. At the same time, she should work to prevent discrimination and exclusion of Ahmed and other foreign students by fostering empathy and understanding in the classroom.

**User:** Would you choose deontological ethics here?

**ChatGPT:** Yes, deontological ethics fits too—acting fairly and truthfully regardless of the consequences. Selma should address Ahmed’s actions while protecting the classroom’s fairness.

**User:** You can choose any approach—situational, social justice, virtue ethics, deontology, or utilitarianism—and give me a clear answer. Which one would you pick?

**ChatGPT:** I would pick Social Justice Ethics. Selma should protect Cem’s rights while also ensuring that Ahmed and other foreign students are not alienated. Balancing fairness and equality supports a safe, inclusive environment for everyone.

**User:** 3. Yasemin, a math teacher, witnesses an incident where her favorite and diligent student, Mustafa, insults Mehmet—who is typically disengaged—provoking a physical altercation. Now, Yasemin must decide whether to discipline them differently. Would it be just to treat them unequally given the circumstances? What would be the right course of action in this situation?

**ChatGPT:** I would choose Virtue Ethics. Yasemin should fairly assess both students’ actions, not their usual behavior. Mustafa’s insult is unacceptable, so he must face appropriate consequences. Mehmet, if he also misbehaved, should be disciplined accordingly. This approach teaches responsibility and supports character development.

**User:** 4. A science teacher notices Gizem constantly disrupting class despite warnings. She knows Gizem has family problems. Reporting her behavior would lead to harsh disciplinary action, but overlooking it would negatively impact the learning environment for the rest of the class. Faced with this dilemma, what should the teacher do, and why?

**ChatGPT:** I choose Utilitarianism—maximizing overall benefit. The teacher should first seek alternative solutions like involving the school counselor to support Gizem emotionally without immediate punishment. Only if no progress is made should the issue be escalated, balancing Gizem’s needs and the class’s rights.

**User:** What would virtue ethics suggest?

**ChatGPT:** Virtue Ethics would advise compassion and justice. The teacher should privately engage Gizem, understand her problems, and guide her to counseling rather than immediate punishment. Supporting Gizem’s growth while protecting the class environment reflects moral character.

**User:** Understood, thank you. Let’s move on to the next question.

**ChatGPT:** Of course! Please share the next ethical dilemma.

**User:** 5. Social studies teacher faces a dilemma: İrem, a hardworking student, fails the final exam. Should the teacher stick to the grade or reward her effort by raising the grade, risking fairness? What’s the right call?

**ChatGPT:** I choose Deontological Ethics. The teacher should grade fairly, reflecting exam results to maintain objectivity and fairness. İrem’s effort can be acknowledged through other means, like awards or praise, but not by altering her academic record unfairly.

**User:** 6. Mert writes a personal essay hinting at past bad habits. The teacher wonders whether to tell the counselor, risking Mert’s trust, or stay silent, missing a chance to help. What should the teacher do?

**ChatGPT:** I choose Virtue Ethics. The teacher should first speak privately with Mert, explain the benefits of professional support, and seek his consent before involving the counselor. This protects trust while aiming to help Mert grow and heal.

**User:** 7. After a tough math exam, most students fail. The administration asks Ali to redo the test, making it easier to appease parents. Ali believes the test was fair. Should he comply or stand by his exam?

**ChatGPT:** I choose Deontological Ethics. Ali should stand by his exam, upholding fairness and educational integrity. Changing the test sends the wrong message about effort and learning. He should explain this to the school administration and his parents, emphasizing long-term student development.

**User:** 8. Emine, a Turkish teacher, observes that Kamil fails his exam, losing his chance to take the national exam (LGS). Kamil is usually successful but had family issues that day. The school has a strict no-exceptions policy. What should she do?

**ChatGPT:** I choose Situational Ethics. Emine should explain Kamil’s special circumstances to the school and advocate for flexibility, suggesting a review of policies for exceptional cases. This way, Kamil’s future is protected while fairness and empathy are balanced. Emine can also suggest broader rules to handle similar situations justly in the future.
